# Perception of students on factors contributing to overweight and obesity among high school students in Kiribati: A qualitative study

**DOI:** 10.1371/journal.pone.0260900

**Published:** 2022-01-20

**Authors:** Tanebu Julia Tong, Masoud Mohammadnezhad, Nasser Salem Alqahtani, Mosese Salusalu

**Affiliations:** 1 Department of Public Health, Ministry of Health and Medical Services, South Tarawa, Kiribati; 2 School of Public Health and Primary Care, Fiji National University, Suva, Fiji; 3 Department of Clinical Nutrition, Northern Border University, Arar, Saudi Arabia; University of Jyvaskyla, FINLAND

## Abstract

**Background:**

Overweight and obesity among high school students is a growing distress not only to the individual wellbeing of a person but also to the productivity of communities and economic expense of both developed and developing nations. This study aimed to determine factors contributing to overweight and obesity among high school students in Kiribati through the perception of students.

**Methods:**

This qualitative study was conducted in four (4) randomly selected senior high schools on South Tarawa, Kiribati from August to November, 2020. A purposive sampling was used to select thirty-two (32) students enrolled into form levels 4–7. A semi-structured open-ended questionnaire was used for data collection using face-to-face in-depth interviews. Data was transcribed and analyzed using thematic analysis method.

**Results:**

This research revealed that the participants were 21 (65.6%) were females and 11 (34.4%) males from form levels four with 9 (28.1%) participants, five with 9 (28.1%) participants, and form seven with 14 (43.8%) participants. Five themes identified including determinants and prevention of overweight and obesity, education and health system factors, stigma, and being fat comes with high risk. These themes collectively elaborate on the essentials of overweight and obesity that are obtained from perspectives of students.

**Conclusion:**

A strong cultural belief and practice has caused misperception of overweight and obesity among students with knowledge-behavior gap recognized as the main reason behind the failure in lifestyle changes among adolescents. Strengthen healthy behavioral lifestyle, improve awareness, and support feasible preventative strategies is recommended to all students.

## Introduction

Overweight and obesity is a major issue that has tripled in the past 30 years [1], across the globe and affects the growth and development of society. According to World Health Organization (WHO), it is the disease of developed countries that has recently become prevalent among Low and Middle-Income Countries (LMICs) especially among 340 million children and adolescents below 19 years of age [2]. Statistics revealed global trend of overweight and obesity among adults to be 36.9% for men and 38.0% for women in developed and developing countries [3]. Prevalence of overweight and obesity among children and adolescents in developed countries revealed 23.8% of boys and 22.6% among girls, while in developing countries the prevalence was 12.9% among boys and 13.4% in girls [4].

Overweight and obesity causes many physical and psychological health challenges among all socio-economic groups, regardless of age, gender, or cultural background [5–7]. Although preventable, the detrimental health consequences of overweight and obesity as known worldwide vary. The psychosocial distress of weight stigma and mood disorder to name a few, together with health impacts of Non-communicable Diseases (NCDs), premature deaths, and disabilities pose risk to the current health of society [8–10]. Since the basis of occurrence is multifactorial, the prevention requires comprehensive strategies and multiple stakeholders to control this widespread public health concern [11, 12].

Statistics from the United Nations International Children`s Emergency Fund (UNICEF) has also reported that 1.2 billion (16%) adolescents make up the world`s population [13]. Therefore, adolescent age group is the target population for this research given the priority in agendas not only in the Pacific region but also internationally. It is a critical phase in an individual`s life where human development rapidly change in terms of physical appearance, behavioural change, and psychological and social development [14, 15]. Moreover, decision making occur and influence adult behavioral lifestyle that leads to unexpected onset of disparaging illness, disabilities, premature deaths, and economic constraint [16]. Preventable secondary cause of deaths due to overweight and obesity is reported among adolescents while alarming rates of avoidable primary cause of death occurs in adult life [17]. This mutual apprehension stresses a fundamental need to explore perspectives of adolescents around this concerning issue.

Overweight and obesity is the major issue in Kiribati where most of its population is facing the consequences leading to the high risk of decrease life expectancy and poor management within the health sector. Reports from Global statistics (2015), mentioned that the highest prevalence of overweight and obesity among Pacific Islands Countries (PICs) is found in Kiribati with 47.7% and 66.1% among boys and girls respectively [18]. Further insights and statistics on this issue will necessitate appropriate strategic measures for health improvements however; there are shortages of qualitative studies in Kiribati concerning this issue. Therefore, since overweight and obesity in Kiribati is on the rise and affecting all generations with different complications, targeting students views implementing strategies early in order to dent the current trend of overweight and obesity that progresses to NCDs and complicates to premature deaths (69%) [19] and disabilities.

This study explored factors contributing to overweight and obesity among high school students in Kiribati, in 2020 through the perspectives of senior high school students and has identified that strong cultural beliefs and practices has caused misperception of overweight and obesity among students with knowledge-behavior gap recognized as the main reason behind the failure in lifestyle changes among adolescents.

## Methodology

### Study design and setting

This study used a prospective qualitative approach where high school students from four (4) randomly selected senior high schools in South Tarawa, Kiribati were interviewed between August to November 2020. This country of study is a resource-limited Pacific Island that lies roughly between Hawaii and Australia and ranks among the highest in NCDs related to overweight and obesity. There are four selected senior high schools represented as School A at the far west, school B and school C towards the central division, and school D towards the east.

### Study sample

This study included all the high school students in South Tarawa. The inclusion criteria included students in Form level 4 to 7, both males and females of Kiribati from four selected high schools. The exclusion criteria for high school students were those who have participated in the pilot study and those not willing to participate in the study. Those who met the study criteria were invited to participate in in-depth interviews.

Since the focus of this study was to explore in-depth understanding of student`s perception on overweight and obesity, this paper followed a method of purposive sampling whereby the sample size of students from each participating school was discussed with experienced principal supervisor and with reference to previous qualitative studies [20–22]. From all consent and assent forms received, the participants were chosen based on research team`s judgment of which students`best fit the research criteria. These students were purposively sampled for maximum variation, availability, and data richness. The students underwent face-to-face interviews until data saturation was reached. A total of 32 students from all four selected schools were eligible to participate.

### Data collection tool

A semi-structured questionnaire with open-ended questions was developed using aims and objective of this qualitative design with clear concept that aimed to grasp first-hand experience of students. The questionnaires consist of two sections: the first part is a demographic summary of the participant with issues relating to gender, BMI-for-age graph according to gender, residing village, and religion, while the second part consist of six open-ended questions on factors contributing to overweight and obesity among high school students in Kiribati. The questionnaire was available in English but was also translated to Kiribati using bilingual translators. This translated version was translated back to English using a different translator to guarantee contents of both English and Kiribati versions were consistent to each other.

### Study procedure

After all ethical approvals and endorsements were sorted and cleared; a trained research assistant was recruited and consented for confidentiality issues to assist in data collection. All senior high school students from the four (4) randomly selected high schools were informed about the research and were all invited to participate. Two sets of information sheets were given, one to students’ age 18 years and above and another to students below 18 years to read and share with their parents or guardians ensuring understanding, confidentiality, and option to withdraw anytime during the duration of research. Two sets of consent forms were also given to students to sign, one to students’ age 18 years and above and another to students below 18 years with an assent form given to parents and guardians of students below 18 years of age. Upon receipt of all consent and assent forms, the date, time, and venue of interview was arranged with the principals and deputy principals of high schools. All in-depth face-to-face interviews took approximately 30 minutes and were handwritten and audiotaped.

### Data management and analysis

The research assistant had audio recorded and manually handwritten face-to-face interviews and sent to primary researcher for transcription. The research assistant verified all transcriptions for accuracy with participants. Upon confirmation of transcription, data was entered into Microsoft Excel where important keywords and phrases were labelled, grouped, and coded with student`s number to prevent duplication. Thematic analysis using Microsoft excel was used as described by Bree and Gallagher [23]. Further coding was done in respect of conceptual framework and research questions by developing a theory and testing the existing concept as described by Young et al. (2020) [24]. All themes and subthemes were identified and formed the basis and results of this study.

### Study rigor

A trustworthy research is considered an appropriate benchmark for evaluating a qualitative study. As Guba and Lincoln (1989) proposed, a research should satisfy credibility, transferability, dependability, and conformability [25]. For the credibility of data collected, three teleconference calls were held for training purposes. Also, pilot interviews among research team were conducted to ensure time-management, feasibility, and comprehensibility. This study has recruited a qualified research assistant with background experience of investigative knowledge, skilful with large datasets, and with multidisciplinary tasks of seniority, nursing, and public health graduate.

### Ethical considerations

Prior proceeding with research, ethical approvals were obtained from the College Health Research Ethics Committee (CHREC) at the Fiji National University (FNU), from the Ministry of Education (MoE) and Ministry of Health and Medical Services (MoHMS) Research Ethics Committee in Kiribati, and Principals and Deputy Principals of participating high schools. The research assistant and all participating high school students were informed of the study purpose, consented for participation, and ensured confidentiality, protection, and security of identities. Informed consents were obtained for the study participants and parents before collecting data.

## Results

### General characteristics of participants

Thirty-two (32) participants were involved in face-to-face in-depth interview. From the general characteristic of all selected 32 students, this research revealed that 28.1% participated from WGMC; the majority of participants were 65.6% females, with highest among 19-year-old students of 34.5%.

Body mass index indicated 71.9% were overweight and 28.1% were obese.

The dominant participating form level was seven among 14 (43.8%) members with dominant religion being Roman Catholic among 40.6% participants with 68.8% reporting both parents being unemployed. (Table 1).

**Table 1:** General characteristics of students (n = 32).

Demographic Status		Frequency	Percent
**High School**	KGV & EBS	7	21.9
	MHS	8	25
	WGMC	9	28.1
	StPC	8	25
**Gender**	Female	21	65.6
	Male	11	34.4
**Age in years**	14	1	3.1
	15	3	9.3
	16	7	21.9
	17	5	15.6
	18	5	15.6
	19	11	34.5
**BMI-for-age according to gender status**	Overweight	23	71.9
	Obese	9	28.1
**Form level**	Form 4	9	28.1
	Form 5	9	28.1
	Form 6	0	0
	Form 7	14	43.8
**Religion**	Roman Catholic	13	40.6
	Latter Days Saints	9	28.1
	Kiribati United Church	9	28.1
	Seventh Day Adventist	1	1
**Working parents**	Father		
	Yes	15	46.9
	No	17	53.1
	Mother		
	Yes	15	46.9
	No	17	53.1
	Both parents		
	Yes	22	68.8
	No	10	31.2

### Themes and subthemes

Five main themes emerged including: Determinants of overweight and obesity, Preventative strategies to overweight and obesity, Health and School related factors, Stigma, and Overweight and obese is unhealthy. The citation of responses uses student`s numbers (S1 to S32), gender (M or F) and age, and school attended. For instance, S3, M18, StPC means student number 3, male of 18 years, and attending Saint Patrick College (Table 2).

**Table 2:** Themes from student`s in-depth interviews.

Themes	Subthemes
Determinants of overweight and obesity	Low income
	Cultural beliefs
	Lack of knowledge
	Peer pressure/ Family influence/ Teacher`guidance
	Screen time abuse
Preventative strategies to overweight and obesity	Individual motivation
	Public awareness
	National support
Education and Health system factors	School curriculum
	School policy
	Health policy
	Trainings/ Implementation
Stigma	People mocking
	Decreased self-esteem
	Psychological issue
Being overweight or obese comes with high risk	Body weight increases
	NCDs increase
	Body`s immunity decreases

#### Theme 1: Factors contributing to overweight and obesity

There are five subthemes identified under the determinants of overweight and obesity. These subthemes included low income, cultural beliefs, peer pressure, too much screen time and lack of knowledge as voiced by students.

*Low income*. Seventeen (n = 17/32) students voiced that living in a family with limited source of income contributed to overweight and obesity in various ways. The low income included having no parents or only a single parent with either a permanent or temporary job. Thus the main contributing factor of overweight and obesity among students in Kiribati were caused by excessive intake of cheap, widely available, unhealthy diet.

*Most times it is the food and drink we have…our family can have donut or bread with tea for breakfast*, *lunch*, *and dinner*. *It is readily available in the cupboard to eat*, *as it is affordable*, *less time consuming*, *and can feed many*. (S1, F15, KGV &EBS)

However, even though increase household members limit the options to food types in every meal, another student commented on the advantage of living with extended families.

*My parents do not work but we are lucky that our extended family live with us*. *So*, *more people*, *more mouths to feed*, *and we can only afford sugar and canned food*, *but on occasions fish*. (S5, M17, KGV & EBS)

The definition of meals to some students is still unclear. Eating junk foods is considered part of a meal rather than dessert. This understanding may be the result of freedom to choose what they want to eat. One student commented on junk food being cheaper than healthy food thus considered an energy consumption diet.

*I stay on Tarawa with my guardians…I hardly eat at home because we do not have enough*. *I eat what I can but mostly junk food at school with my friends and this is what keeps me going every day*. *Bongo 50 cents and is way cheaper than an apple which cost $1*.*20 for a small one*. (S10, F16, MHS)

Healthy foods on South Tarawa are costly since the majorities are produced with tremendous time, effort, and risk. For instance, since soil is brackish on the island, vegetables are grown with sweat and energy. Even fishing is risky as many fishermen are not fully geared but still go out to fish for food or to generate income.

*On South Tarawa*, *life is expensive compared to outer islands*. *A fresh 10 pounds tuna cost $15*.*00 on Tarawa while it cost $6*.*00 at outer islands*. *But I come only for school and trying not to take things for granted*. *Healthy food is awfully expensive*. (S11, F15, MHS)

Another student commented that living on the capital island is more complex than living in the outer islands especially to those with low income.

*No work is a problem on South Tarawa but in the outer islands*, *fishing and planting can keep a whole family filling and at peace…uh*, *but here at school*, *I eat anything that is edible with friends*. (S15, F15, MHS)

The understanding of urbanization brings about change in an adolescent`s life. It is the period where their knowledge is put into practice, but the surroundings become stronger influences on their lifestyles. Since low income plays a significant role in contributing to overweight and obesity as claimed by a few students, one student voiced that behavioral change has placed income and skills in the wrong places at the wrong time.

*My father can fish and plant when we lived in the outer islands*, *but now he spends more leisure time at a kava bar at night and sleeps during the day*. *So*, *we end up buying affordable food only*. (S17, F19, WGMC)

Evidently, overweight and obesity depends on socioeconomic background of families. Cheap, unhealthy, and readily available meals are easier to buy, to feed an overcrowded home. Moreover, behavioral changes also impact overweight and obesity. For example, free time is poorly managed as more heads of family spent time with friends drinking kava at night and sleep during the day. Thus, result in the consumption of un-nutritious diet by children and family members.

*Cultural beliefs*. Culture and practices in Kiribati are extraordinarily strong especially when talking about physical appearances. From infancy, children are fed frequently and massaged regularly for muscles to start developing early. As they grow into childhood, the consequence of massage and habit of frequent meals persist as norms. Once a child reaches puberty, the females undergo strict diet (water and coconut) for three days during menarche with abdomen tightly wrapped with strong locally made fiber rope while the boys feast well for three days after circumcision. These two cultures still exist and believed to being the main reason behind physical appearance and dietary habits in Kiribati.

Since, the cultural norms and beliefs greatly influence the upbringing of children and such is seen during adolescent years. Seven students (n = 7/32) mentioned local massage and partial understanding of feeding methods to the young. During childhood years, especially females, parents continue the legacy of local massage for children to become big and chubby as it is always a belief that big is beautiful in most countries around the Pacific. Even the amount of food introduced to toddlers is way more than the normal amount of diet. The outcome of food consumption and massage is overweight and obesity.

When asked to comment on the eating habits seen at home, school, and community gatherings, one student mentioned that eating more without exercising becomes a norm especially when the weather is not favorable for physical activity.

*Overeating is a norm…one plate of food does not satisfy my tummy…uh; I do not have time to work out after eating as the weather is extremely hot and it makes me feel tired*. (S2, F15, KGV & EBS)

Moreover, it is the consequence of cultural practices that also play a role in big body size among adolescents. Students appear well versed around the concept of cultural beliefs and practices.

*All my sisters are chubby and so I think it is the outcome of my grandmother`s massage skill when I was a child…umm*, *now for me*, *I cannot think of a plan to be fit as it is difficult to find motivation*. (S13, F17, MHS)

It is obvious that students continue to believe in cultural practices but motivation to prevent the consequence is absent.

Two students explained the culture behind infancy massage, menarche diet in females, and feasting nutrition in circumcised males.

*It is funny how culture is a question nowadays but back in time it was pure identity*. *Long before*, *my grandfather always explained that the practices were normal because the load of work was heavy and the body expenditure on energy was well used*. (S5, M17, KGV&EBS)

Another student elaborated on practiced culture and stated,

*My grandmother believes that the story behind the menarche in girls and circumcision in boys is back in those days’ men were warriors fighting for land during the pre-colonial days and therefore their bodies need to be always strong and thus feed well*. (S9, F16, MHS)

It is also wise to learn the culture in school as shared by one student praising the lessoned learned are put to good use.

*I studied in history class that females have strict diet during menarche because it is a time they enter adulthood and should know their position in a household where they maintain their physical curvature while men build up muscles*. (S17, F19, WGMC)

Moreover, learning from experience is another way to hold steadfast things that has happened. As elaborated from one student the experience of 8 years back.

*From experience*, *the menarche diet is supposed to teach females how to control eating*, *however*, *in my experience*, *it is a total opposite*. (S22, F19, WGMC)

Most importantly lessons taught from elderly grandparents is one to treasure as they are primary resources that have experienced life for long and because culture and practice is slowly diminishing, and no resource is available for rejuvenating the past. One student proudly tells of the teachings of an elderly grandparent.

*My grandmother taught me culture in many aspects*, *from black magic to physical appearance*, *and love*. *I remember the explanation behind the females menarche diet was building curvature in a woman`s body or what others sometimes call a figure of number 8*. *It is meant to be big in the upper and lower body for strength and power to be able to carry out the duties of a mother*, *household chores*, *and a wife without assistance and complain*. (S27, F19, StPC)

*Peer pressure or role models*. Interestingly, six (n = 6/32) students claimed that eating with friends or families result greatly in overweight and obesity. Having someone to talk to over a meal or snack lengthens eating time and moreover, results in too much relaxation. Furthermore, smoking, drinking alcohol and kava is tempting when students plan a function.

Considering overweight and obesity, students were asked to comment on colleague’s attitude and practices. However, the comment received from one student is that eating together with friends is always fun and enjoyable. Maybe the excess consumption of food without physical activity among friends’ influences weight gain during high school days.

*High school life is fun because we can enjoy each other`s company while sitting*, *eating*, *and studying…I don`t think I like any physical activity and luckily*, *sports is not compulsory in high school*. (S13, F16, KGV & EBS)

High school life to some students is always about enjoying each other’s company and physical inactivity but it is also a place where alcohol and other substance influences and experiences begin. Students highlighted that peer pressure is the most influential in lifestyle behavior of adolescents. One student commented,

*Drinking alcohol for the first time was with friends…they were having a blast and I thought I should join and experience once*, *but it seems like a habit now*. *I know alcohol contributes somehow to overweight and obesity*, *but it still tastes good*. (S3, M16, KGV & EBS)

Another student stated that seeing friends enjoy drinking kava was a temptation without considering the consequence. This is the logic behind the temptation from his perspective.

*Because many of my friends enjoy kava…I did not think it would impact anybody change*, *but I guess the overnight drinking*, *the aftermath sleep*, *and the sedentary lifestyle does impact overweight and obesity*. (S20, M19, WGMC)

Role models like family members; especially parents almost always set examples for children and adolescents in their households. Family influence is particularly as important as peer pressure and teacher influence. One student highlighted the influence of family in his lifestyle.

*Every day*, *life at home is relaxing*. *My parents are big people*, *and I am also big in size and eating is like a sport in my home without much exercise*. *This study however has made me visualize how ignorant my family and I have been in terms of body size and the risk we possess*. (S4, M17, KGV&EBS)

Another student was more positive in his comments and said,

*I do join my teacher in weightlifting workout*. *I am now overweight in BMI*, *but I do hope it is the weight of the muscles I have developed and not fat*. (S5, M17, KGV&EBS)

Apparently, influences from friends, teachers, and family members can be turned positive if adapted into strategic measures.

*Screen time*. Fifteen (n = 15/32) students mentioned that studying, watching movies, and use of mobile phones take up most of the hours of the day. Screen time the whole day as mentioned in the interview is a significant contributor to overweight and obesity. One student mentioned the number of hours spent on screen is distracting if fully calculated.

*First*, *I attend school from eight o`clock in the morning to four o`clock in the afternoon*. *Then I take afternoon naps and do few house chores*. *At night I spent most hours on screen whether it is for study or relaxation…but I waste too much time on social media*. (S26, F17, StPC)

When students were asked to comment on the use of electronic gadgets, one student eagerly mentioned,

*I rather stay indoors on screen than move around outside in the hot weather*…*there is nothing fun to do so I spent hours and hours chatting with friends online*. (S22, F19, WGMC)

Definition of fun and socializing to some students is all about surfing the net or chatting online. One student voiced with some common sense that although social media is fun, it is overriding time management.

*During my spare time I play games online*. *This is supposed to be for relaxation*. *But I think it is using up most of the hours in a day*…*and cannot really manage my time*. (S32, M18, StPC)

Seemingly, screen time is on the rise with detrimental influence on adolescents not only psychologically but also in terms of physical inactivity and outlook of one`s body.

*Lack of knowledge*. Lacking understanding of the causes and outcome of overweight and obesity among adolescents is common in Kiribati as voiced from nine (n = 9/32) students. These students mentioned that many of their parents did not understand anything about overweight or obesity.

*When I tell my parents*, *I will cut down on eating*, *due to overweight and obesity*, *they tell me that I will get sick…and therefore since support is minimal*, *enthusiasm on the topic and issue diminishes*. (S30, F19, StPC)

One student explained specifically that his parents are not aware about the concept of good nutrition.

*When I tell my mother about eating dried salted fish everyday then she comments*. *What is wrong with dried salted fish*? *I am who I am today because of this diet*. *Then what diet do you expect to eat*? *Chicken*? *Ham*? *With colorful beans for decoration*? *Do not blame the food I prepare*, *blame your freedom in lifestyle*. (S30, F19, StPC)

When trying to enlighten parents or grandparents on what is taught at present in schools, being told off is the response believed to happen.

*I told my father that sedentary lifestyle will worsen our family condition since both his parents passed from diabetes…mmmm*, *did I just bought myself an airplane ticket to go live elsewhere*. (S25, M19, StPC)

Knowledge in Kiribati is a one-way street for many families and part of the reason being is culture. No child can comment to anything an older person says or does. Today, human rights have changed the perspectives of younger families but to the older generation, the teachings and practices of the past remains the same.

*I know that culture is my identity and that should never change*, *however behavioral lifestyle is not an identity to my ethnic background and therefore anything educational and healthy*, *I try and practice and say at home*. *It is an advantage of being the only child where my parents…in their 50s… although extremely strict*, *they always have time to discuss and share my experience and compare with their own…it is slowly turning the hands of their clock into understanding modern life more fully*. (S15, F16, MHS)

Another student mentioned that their parents continue the legacy of big is beautiful in the Pacific despite the detrimental health consequence. However, some parents lack health aspects of big body size and do not believe in learning from the younger generation.

*My parents are not aware of the concept Big because in their eyes*, *it is beauty and strength*. *It is just after providing them with the information sheet that they ask occasionally why overweight is not good*? (S13, F17, MHS)

The response, how many coconuts have you eaten before me is a metaphor occasionally used by the elderly generation especially when they refuse to accept an explanation from the younger generation. It generally refers to the number of years of experience between the two parties in conversation.

*My mother tells me that I must eat more to be beautiful and masculine*…*well I replied with a long explanation on the difficulty of energy expenditure that will be stored as fat…um*, *the reply I got was*, *how many coconuts have you eaten before me*? (S28, M19, StPC)

Lack of knowledge is indeed a barrier to overcome for everyone to have a mutual understanding of the concerning issue.

*The information sheet of this study shared with my parents was exciting because issues like overweight is the main cause of discussion at home*. *My mother tells me to share all that I know on this topic because it is the first time; they hear that it is a risk to unwanted complications*. (S13, F17, MHS)

#### Theme 2: Associated factors to overweight and obesity prevention

This theme identified three subthemes that include individual motivation, public awareness, and national support. Prevention as known from previous research and this study is cost effective rather than waiting for cure or therapy. Moreover, the burden of prevention on an individual is less disheartening rather than the heartbreaking effect of overweight and obesity. The students interviewed expressed their perspectives on ways to prevent overweight and obesity while still young.

*Individual motivation*. Foremost, any change required to an individual behavior requires self-motivation and implementation. However, twelve (n = 12/32) participants interviewed stated that individual motivation is lacking as many students lack time management. One student sadly voiced the stress she is undergoing with disorganized schedule and thus not enough time to work out.

*I think my time management and focus is lacking because every day for me is exhausting despite not being physically active*. *I cannot really finish required duties on timely basis*. *My schoolwork is already stressing my focus*. (S18, F18, WGMC)

Another student highlighted the need for group effort to accomplish a goal and she mentioned in the interview,

*I know I can exercise and eat less but I want to do it with other friends*. *I need to find someone who can inspire me to work out every day because it does not occur to my mind the need to do so every day*. (S7, F17, KGV & EBS)

Motivation is required from every aspect available. Some students emphasized the need for time management while others recommended team effort to combat any obstacle. However, another student requested the need for a coordinator with background profession in physical health and sport.

*We need an instructor who can motivate students to work out and eat less…oh*, *and the drinking of water is difficult to even think about because access to fresh water is limited on the island*. (S9, F16, MHS)

One student voiced that change has to come from self-first before attempting to change others. He mentioned,

*Like any other issues concerning an individual*, *any change to anything must come from an individual’s will and determination*. *Although many students may believe in the need of motivation*, *the individual self-respect should exist to aid the work of a motivator*. *This is an exceedingly difficult concept for many to grasp because everyone in Kiribati is spoon-fed*. *Rarely*, *I see friends experience on their own*. *It is either with others or not at all*. (S4, M17, KGV & EBS)

Another student claims a one-way street in his intellectual thinking. He states that he can only accommodate one goal at a time.

*I think if I am motivated to something new*, *my focus in school or life diverts*. *Maybe it has to do with how I was brought up…um*, *I believe it is lacking determination*, *but my parents call it procrastination and peer pressure combined in my mind*. (S3, M16, KGV & EBS)

Sometimes the definition of motivating self is deemed gloomy when there is no self-esteem. It is the worry of other`s perception on what you really want to accomplish. One student stated that being fit is not a problem but maintaining fitness is a worry.

*Funny*, *that all these years I have groomed myself looking healthy and fit*. *After participating in this study*, *I have just realized that I need to change my vision of what health really is*. *I can start exercising but just afraid of what other will say*. (S24, M16, WGMC)

Some students understand the disheartening consequence of overweight and obesity; however, the implementation of preventative strategies brings about stigma. One student mentioned in her comment,

*The complications of overweight and obesity are hard to imagine*. *However*, *how can I change without people noticing*? *Just petrified by incoming comments from friends and the public*. (S26, F17, StPC)

Another student elaborates more on implementation approach with additional comments on personal thoughts.

*Just having the will power*, *self-respect*, *and determination will boost our individual motivation to being healthy*. *No need to worry about what the public thinks*. *It is just what I believe*, *however*, *the implementation of such is a different story*. (S14, M18, MHS)

Specifically, individual motivation as the two words implies must come from within. It truly requires self-worth, determination and will power as fully understood by some students but stigmatization that follows will be dealt with at a more community level.

*Public awareness*. The public`s understanding and knowledge on overweight and obesity will lighten the works of prevention. Having a single goal with the public as a team will ease any build up tension. Thirteen (n = 11/32) students had different perspectives and views on overweight and obesity prevention.

Since the basic knowledge and understanding of overweight and obesity is the work of MoE incorporated with MoHMS, one student requested assistance from the health sector to meet with communities to promote awareness on weight gain and its complications.

*I wish there is a qualified trainer in school that can coordinate proper times for training and who understands the issue of overweight and obesity among high school students*. *In addition*, *MoHMS should promote awareness and preventative strategies on overweight and obesity in communities*. (S29, M19, StPC)

Similarly, another student expressed that change needs to be tackled from every corner of civilization.

*We need our health team to be at every corner of society from the very start to change the perspectives and behavior for the future*. *Education teams also need to do the same but in schools*. (S31, F19, StPC)

Meanwhile, one student mentioned that, changing the face of society for the better requires long-term public awareness that focuses on the upbringing of children.

*It is hard to change the behavior as we mature so if the public can be encouraged to start earlier with children*, *eating healthy and physical activity are the focal reasons to being fit*. (S5, M17, KGV&EBS)

Additionally, the food portion, preparation, and nutrient in family meals requires attention as many students claim that healthy foods are expensive, but reality speaks for itself when many parents and guardians afford smoke, alcohol, and kava on daily basis as indicated by one student.

*The main problem is the diet*. *There is no appropriate food quantity in family meals*. *It is all about eat as much as you can*. *Eating meals is all about Carbohydrates and Protein*. *Fruits and vegetables are hardly seen as part of regular meals*. *Moreover*, *they say it is expensive but you still see people affording to smoke*, *drink alcohol*, *or kava that are more expensive fruits and vegetables*. *Public awareness is a must*. (S23, F19, WGMC)

Moreover, one student expresses the need for health to utilize community gatherings for public aware programs on overweight and obesity. She states,

*Since communities like to socialize*, *the health should make efforts to visit on regular basis for both the motivation and awareness on overweight and obesity cause and effect*. (S15, F16, MHS)

Student`s concerns on preventing overweight and obesity were raised with relevant points. Looking at health visits to all levels of primary prevention starting from homes, churches, and schools may impact behavioral change significantly. There is no harm in starting at the very beginning where one student raised her concern and suggestion.

*I am not worried about physical activity; my worry is the diet my people are used to*. *Since childhood*, *the diet has been so wrong*. *Feeding until full is the understanding rather than feeding with the right quantity*. *Having health talks by students to communities is another focus to consider*. (S17, F19, WGMC)

Public awareness was eminent among responses given by students and could be the access to overcoming barriers concerning societal issues.

*National support*. Fifteen (n = 15/32) participants interviewed voiced that the government and stakeholders should be proactive in the prevention of overweight and obesity. Funding, extracurricular activities, and infrastructure need to be well organized and maintained according to societal changes. The government needs to set priorities and goals according to the response of its people and not continue to spoon feed until who knows when.

It is impractical to know that the government supports those early high school dropouts financially. However, one student voiced concern on this financial aid.

*I thought the government was poor but lately*, *those who fall within the age of 18 to 59 years without jobs and not attending school get financial assistance of $50 per month*. *Seriously*? *What will this aid counteract*? *It is enough to spend overnight drinking kava*, *buying smoke*, *or drinking alcohol*. *I prefer more government-sponsored gymnasiums are built for physical training indoors*. (S8, F16, MHS)

Another student raised concerns in terms of marketing costs. Although some fruits and vegetables were produced locally, the cost of market is extremely high.

*The government needs to subsidize the cost of fruits and vegetables… it is awfully expensive*. *One whole watermelon cost up to $60 Australian currency at $15 per kilogram*. *The watermelon is from one local farm on the island*. (S5, M17, KGV & EBS)

Apparently, voices raised from the students, request the need for more training schools as a strategy to combat financial difficulty that may interrupt the current overweight and obesity trend.

*If the government can provide vocational schools for those facing financial difficulties*, *it will become a blessing*. (S12, F14, MHS)

One student even expressed the perspectives on activities and the sponsors that have happened in communities.

*If stakeholders can invest in supporting students into schools rather than sponsoring for talent shows and beauty contests*, *the outcome could be for the better*. (S10, F16, MHS)

Interestingly, with fancy quotations, another student emphasizes the need for government to support health and education sectors for quality measures.

*Knowledge is power*, *strength is health*. *These are the qualities that are necessary in life*. *Would love to see the government maintain support in health and education*. (S16, F18, WGMC)

The government is requested to show full support on the issue around overweight and obesity, as most determinants are modifiable.

#### Theme 3: Education and health system factors

This theme managed to identify four subthemes that comprise school curriculum, school policy, health policy, and trainings/ implementation. Education and health is essential for prosperity of society as mentioned by students interviewed. Especially when concerning the physical wellbeing of an individual, there should be a mutual understanding of what needs to be done and this all depends on the trainings, the guidelines, and implementation.

*Curriculum*. Physical Education starts from primary years but somehow escapes the requirements during adolescent school days. In Kiribati, physical education in high school is optional as the main teachings are focused on concept. Five (n = 5/32) students voiced the need to review the curriculum for education from primary to secondary levels. One student commented on the physical wellbeing during primary days compared to high school times.

*I know I was physically fit before but now I can feel that I am easily tired and lazy to move around*. *I spent hours completing assignments and I sit almost the whole day in school*. (S15, F16, MHS)

Another student highlighted the need to study without distraction as scholarships during senior high school days is competitive.

*School is competitive especially when applying for a scholarship …and so I focus on reading and writing only*. *I do not have time to play sports*. (S19, F18, WGMC)

Moreover, several students are requesting physical education be a part of high school certification.

*The only sport I find time to play in is volleyball*. *I play with friends to socialize*. *It is not on regular basis but only on weekends*. *I hope physical education is back into high school curriculum*. (S17, F19, WGMC)

When high school students were asked about sports or physical education in their curriculum, one student responded.

*Playing sports in most high schools is optional…few students play together to sweat out the stress…but the majorities just sit around telling stories while some still find time to use their mobiles against school rules*. (S27, F19, StPC)

The curriculum in high schools is focused towards scholarship requirements. It is a competition among students. One student mentioned that physical activity is now not a major concern but the theory and academic know is.

*…the main focus in high school now is getting a scholarship*. *There is no time to play or waste time…uh*, *high school life is both fun and stressful*. *I need to focus on study and that also requires a lot of late night snacks*. (S26, F17, StPC)

One student highlighted his interest in sports and wished it would become part of the scholarship offer in the future.

*There should also be a scholarship for sports*. *Maybe that will also trigger the interest to physical fitness*. (S14, M18, MHS)

Meanwhile another student expresses opinions on having both, physical education and health, as elective courses in high school since overweight and obesity evolve around these general science topics.

*Diet is the main problem*. *How to eat*, *what to eat*, *and amount to eat should be shared with students and the communities we live in*. *It is good to start from our very homes*. *Specifically*, *elective courses on nutrition and physical education should become part of the school curriculum*. (S31, F19, StPC)

Putting health and physical education in the current curriculum is the issue raised by students. Each student knows and understands about overweight and obesity but the actions and practices towards the knowledge require motivation.

*Policy*. Four (n = 4/32) students interviewed stated that policies in schools are dusty and needs polishing. This means that many policies are outdated and requires review to current standard of living.

*I am not aware of any health policies in school because there is hardly anything on the shelf of our school office*. (S17, F19, WGMC)

One student voiced part of a health policy that is repeatedly mentioned and highlighted to students concerning good hygiene.

*Nothing is ever shared in school concerning health status*. *The only thing you always hear about is the good hygiene practices*. (S11, F15, MHS)

When policies are available, the implementation is lacking as voice by one student.

*There are school policies but all concerning other issues like transport*, *alcohol*, *kava*, *and smoke*. *There is also a food policy but not so much implemented by school canteens*. (S22, F19, WGMC)

Only a few responses were received from students for policy understanding thus this signifies little interest and concern on the foundation of every functional institution.

*Trainings/ implementation*. Maintenance of policies, buildings, and equipment are exceedingly difficult among the Kiribati population. It is their mentality of wanting to get new things and more training but not so much maintenance and implementation. Two (n = 2/32) also voiced the importance of implementation in the prevention of overweight and obesity.

*There may be policies lying around in school offices but the there is no implementation done in schools*. *More policies concerning school students and health are recommended*. (S27, F19, StPC)

Although Kiribati is a resource limited country, a student highlighted that there are other ways to overcome financial crisis and that is to begin with personality change.

*We are not a rich country and should progress with what we have or able to get*. *If we are in financial crisis*, *we should understand our own needs and prioritize over our wants*. *This way we can manage to see if we can afford an apple rather than alcohol*, *or do more physical activities and reduce screen time*. (S31, F19, StPC)

#### Theme 4: Stigma

The three subthemes involved in this theme stigma are people mocking, decreased self-esteem, and psychological issue. Stigma is common in the Pacific and worse in Kiribati. The public will always find any reason to laugh or giggle despite the tension in any case scenario.

*People mocking*. Imitation of any sort decreases motivation to accomplish a set goal. A quote coming from a male student said,

*I was trying to lose weight one time and jogging was in mind*. *However*, *while jogging*, *I received comments from the public like*, *oh he wants to be like a foreigner or how can he exercise when he is so big*. *This decreases my confidence to continue exercising*. (S25, M19, StPC)

It is a habit in Kiribati to mock and gossip about nonsense things. However, this behavior is influential in demoralizing one`s goal of being healthy.

*I cannot exercise because it will mean that I am weak in facing the consequences of obesity*. *This is wrong concept in the history of health*. *What shall I do*? (S5, M17, KGV&EBS)

Any comment from the public is a strong voice and weight to ones ear because in Kiribati, there is a saying that news travel fast through coconut wireless connection. This is meant by the gossip that travels in the air. What is said or laughed at in one end get to the other end in the very next minute or two.

*I have an older sister that was got divorced because her parents in law saw that she is getting big*, *lazy and may be requiring a lot of food to eat…so she got sent home*. *This is sad news to my sister and her husband because my sister was pregnant*. *The reason her parents in law despise her is because of hardship in the family but other families members and neighbors laughed and commented that it was because she is big*. (S1, F15, KGV & EBS)

One student commented on the need to work out due to big body size but is reluctant to do so when thinking about the public viewpoint.

*The eye of the public makes me feel uncomfortable when I start working out because I am beginning to feel heavier and lazier doing schoolwork*. (S21, M19, StPC)

When the islanders say that laughing is a medicine, one student commented on a wrongful act that suddenly realized.

*Laughing is a medicine they say*. *When you laugh that means you are happy*. *So even if nothing is funny*, *others will find comments to make others laugh*. *One boy was big but we see him eating that made the rest of my friends laugh*. (S16, F18, WGMC)

Joining into something just because you want to show your friends that you are still on their side will get people in trouble.

*Laughing shows that you are part of the group*. *One time I got scolded by my big brother because my friends and I mocked him for not being able to climb a coconut tree because he was big and my father had to do it*. (S22, F19, WGMC)

*Decreased self-esteem*. This issue is common among most students interviewed. It is a cultural norm to be shy in Kiribati and every aspect of mocking decrease self-esteem more. One student explained with shyness that,

*It is difficult to accommodate exercise and healthy diet in Kiribati because it is a western lifestyle…in Kiribati we are used to doing traditional chores as exercise and local foods as healthy diet*. *This is seen back in the outer islands but not on the Capital Island thus my motivation to local lifestyle is diminished here on the main island*, *this is only my view*. (S18, F18, WGMC)

Self-esteem and self-confidence are not taught but acquired during upbringing. Corporal punishment was once a way to teach the islanders of what and how things should be done.

*I am more mature than most students in my class*. *But still the shame of physical appearance weakens my confidence*. *It is sad when I want to play basketball with friends*, *my parents say it’s a waste of time*. *It is funny that I see younger friends being allowed to play when even I am afraid to play without proper approval*. (S27, F19, StPC)

Moreover, doing work in a group builds confidence in older generations because if anything goes wrong, they will share the punishment.

*I can join friends running on school campus but I cannot run alone because I am afraid of any unforeseen trouble that may happen*. *If I have friends around*, *then at least one or two can be witnesses or immediate helpers*. (S32, M18, StPC)

Another student requests assistance in finding the appropriate and attainable method to this concerning issue.

*The main concern is being overweight or obese*. *This issue already decreases self-esteem and self-confidence…I really need strategies to overcome this problem as a whole…please help find the most fitting approach*. (S12, F14, MHS)

*Psychological issue*. Stress is the common issue around this topic of interest. Many students expressed that schoolwork stress and other relationship stress has increased the tension around food cravings. Some students’ highlighted friends going through similar situations and at times they feel like following in the same path.

*I feel that I am eating more because of stress and moreover depression on my academic performance*. *This has been my coping mechanism since I have started high school*. *Now it is coming towards the end of high school days and feels my effort is just average and this is not enough to compete in scholarships*. (S17, F19, WGMC)

These are examination years for scholarship, already stressing out students. One student commented on stress and food.

*I need to study for scholarship exams and food has been my lifesaver throughout*. *Although I am big in size*, *I cannot imagine life now without my love for food*. (S26, F17, StPC)

Boyfriend/ Girlfriend relationship and breakups both influence food cravings one way or another. One student regretfully stated,

*I am big and healthy and was in a relationship until the point I had to manage my time for studies*. *I thought that being in a relationship was the reason behind my body size*, *but it seems like breaking up worsens the situation while stress in studies builds the tension*. (S13, F17, MHS)

Another student stated that life is complicated. How to deal with the situations faced is more concerning and requires immediate support.

*I am obese and this is the way it has been*. *It stresses me to even think about the ways to decrease my weight without anyone noticing*. *I believe the only way to solve this issue is either to return to the outer islands and live a local lifestyle or remain on the main island and face it one day at a time*. *I have decided to stay firm and hope for change soon*. (S14, F18, MHS)

#### Theme 5: Being fat comes with high risk

This last theme has recognized three subthemes such as body weight increase, NCDs increase, and body`s immunity decreases. Big in the Pacific context is beautiful however, big in the health framework is a risk. Students understand both circumstances and wishes to contribute to change if support is available as most students’ voice both perceptions and desires.

*Body size increases*. The word fat is harsh and gloomy, however overweight, and obese is more a technical word for it. One student commented,

*When people say I am fat*, *I get depressed and feel sad however*, *hearing the word overweight and obese I do not feel disheartened…so why this confused feeling*? *I just need motivation and support for behavioral change…if not now then for the future generation*. (S4, M17, KGV&EBS)

Some students mentioned that since entering high school, the weight increases. Little did they know that they have been physically inactive throughout the high school days.

*I thought I was between normal weight ranges but now I know that I am obese*. *I have reflected back since entering high school and noticed that there has been no physical education class*. *This is unbelievable how I have overlooked and it did not click my mind until now*. (S14, M18, MHS)

Many things have increased adolescent`s body weight. Anything from small to big the chances to increased body size is high.

*I have this obsession of eating junk food during study time…it keeps me awake and energetic*. *The consequence however has been overlooked*. *This study has helped me look at the outcome from a different view*. *Now I will seek cost effective programs on how to change this habit and issue of being big*. (S20, M19, WGMC)

*NCDs increase*. Many students voiced a lot of responses on overweight and obesity with NCD relation. This emphasized that knowledge is already available but the requirements to completing the cycle is recommended.

*I understand that NCDs are increasing and the main contributing factor that is approachable is overweight and obesity*. *This NCD is claiming the lives of young people and heartbreaking to their families*. *I am also obese and it scares me to even think about the risk I have towards NCD*. (S18, F18, WGMC)

Another student looked at NCD from a preventative measure and states,

*NCDs are shortcomings of individual*, *education*, *and health preventative strategies*. *I know that Ministries of Education and Health are working full time to combat this NCD…but we*, *the people*, *are not doing much to support the works of our government*. (S31, F19, StPC)

One student looked and commented both ways to combating NCDs among students.

*It should always be a win-win scenario with any issue or concern battled*. *I believe that the issue here is overweight and obesity that is contributing to premature deaths and disabilities among the population*. *We need to combat overweight and obesity from every corner possible*. *Since high school students are the targets at this time*, *I challenge all adolescents to ask the question*, *what I can do now to combat overweight and obesity*? (S32, M18, StPC)

*Body`s immunity decreases*. Immunity decreases in many aspects of health disease. Overweight and obesity is a gateway to this immunity suppression in our individual body. One student commented on his family member and relates to the current situation.

*My grandfather is obese*, *she is also diabetic*, *I know that any flu or communicable illnesses that swings by*, *he gets*. *This is sad because I am also obese but at an incredibly young age*, *if I do not do anything now*, *I believe I will fall in the footsteps of my grandfather*. (S28, M19, StPC)

Another student expresses the fretfulness on the issue and visualizes the endpoint of the disease. This student also leaves a similar question everyone else has been asking.

*Now I see myself as overweight and obese but I cannot imagine how I will be with NCD and suppressed immunity*. *All this issue about big is beautiful will all end up in a box early*. *I need to change now but how*? (S1, F15, KGV&EBS)

Hospitals are also overwhelmed with patients having suppressed immunity and many deteriorate fast when not treated on time. One student commented on this issue.

*I have assisted my aunty who is overweight and has diabetic foot ulcer*. *I nearly lost this young aunty because she has decided to use some local medicine for her ulcer instead of being seen at the clinic*. *The foot ulcer got worse and when all seemed hopeless*, *my family decided to take her to the hospital*, *we were late for treatment*, *her immune system was weak*, *and the best option was cutting off her foot…incredibly sad but true*. *I feel scared knowing I am obese*, *and I do not want to see part of my body gone just for being ignorant*. (S4, M17, KGV&EBS)

## Discussion

Some important facts gathered were the perspectives of students in terms of causes and consequences of overweight and obesity. It was collected that most students that participated had the knowledge and understanding however, it seemed evident that such information is not being transferred into actions and practices [26, 27]. Further analysis of the results has pinpointed possible reasons behind the knowledge and performance gap among adolescents in Kiribati.

The cluster of viewpoints obtained by student were the determinants and preventative strategies to overweight and obesity, the education and health systems, stigmatization, and being fat comes with high risk.

### Determinants of overweight and obesity

Low-income status has prevented the implementation of knowledge attained by students in terms of maintaining healthy diet. Since the marketing costs of fruits and vegetables have increased locally, those students with unemployed parents are deprived from getting the nutrition their bodies require. Moreover, parents with low income may not have transportation to buy fresh food and furthermore, the shops may not be within walking distance. This is also highlight in the studies done by Lavine (2011) [28] and Phipps et al. (2006) [29] where both studies stated that obesity is more prevalent among those with low-income status.

Secondly, the cultural beliefs and practices have influenced overweight and obesity one way or another. The former idea of big is beautiful [30], cultural value in infant`s body massage, and feeding practices have inspired such drift from the past. Although adolescents have the power to make choices of their own [31], the upbringing has set pace especially in diet and exercise and therefore interfering with the individual behavior. This will require cultural transition to progress with weight loss programs. As also revealed by Diaz (2007) that accepting preventative measures for overweight meant shift in cultural norms [32].

Lack of knowledge is referred to parents`or grandparents`perspective on cause and effect of overweight and obesity. This possible factor could be related to cultural norms and practices where many elders refuse to accept the current enlightenment on high-risk issues. Dunifon (2012) specified that grandparents do not influence adolescent’s lifestyle, as past practice is different from current situation. Furthermore, parents and grandparents rely on past experiences and thus continue to practice at present.

Peer pressure and other related influences were also highlighted. The majority of influences on adolescent’s lifestyle were mostly peer related however, as detailed in Peneau et al. (2009), eating with friends in groups increases food consumption [33]. In order to understand the lifestyles of adolescents, the results also elaborated that students learn and practice what they see and experience in life through friends, families, teachers, and other mentors, in which according to students, their role models. Cruess et al. (2008) also cited, role modeling is the most powerful teaching strategy proven effective [34].

Lastly, the amount of time student’s use on the electronic gadgets is another determinant of overweight and obesity. Mitchell et al. (2013) identified that screen time is a known risk factor to weight gain [35]. The reasons to increase in screen time are vast and addictive thus interfering with daily normal practices of healthy eating and physical exercise [36, 37]. Social advertisements also increased cravings for junk foods another issue to consider as stated by Pearson and Biddle (2011) [38].

### Preventative strategies to overweight and obesity

The development of common interventions through Individual motivation, Public awareness, and National support were key findings identified for preventative measures.

Interventions targeting individual students tend to collapse as influences from friends, families, and teachers tend to triumph [39]. Thus individual motivation from personal to community levels is voiced and recommended by those victimized by the overweight and obesity issue.

Public awareness is another recommendation for combating overweight and obesity as this study and a study by Hooper et al. (2017) highlighted [40]. It is the people and their environment that needs to be acknowledged and understood for such strategy to work [41]. As responses illustrate, the public have their own socio-cultural norms and any disturbance will damage societal behavior towards proposed interventions. Therefore, considering the practicality, sustainability and most cost effective method should be the strategy of interest [42].

National support as mentioned by Tremmel et al. (2017), the economic burden of obesity has called for public health measures [43]. Most times the weather is not promising as it can be either too hot or too wet. The need for indoor gymnasiums with appropriate equipment and qualified facilitator, new policy aimed at healthy diet and physical endurance in schools and workplace, and furthermore, scholarships for sports and nutritional health post high school. It appears that there is still need for government support within this area but requests are intended for multidisciplinary approaches that are adequate and sustainable.

### Education and health system factors

Overweight and obesity is common among adolescents and multiple studies have recognized schools as the fundamental setting behind health promotions in preventing the progress to complications and Bleich et al. (2018) believed in the same concept [44]. Since students spend more hours in schools if not at home, then school teachers and facilities can assist in solving the overweight and obesity epidemic. Studies done by Centeio et al. (2018) [45] and Manios et al. (2012) [46] elaborated on education as being the first step towards preventative strategies. However, this study also emphasized that sturdy school-based policies, effective curriculums, and experienced mentors are advocated to disseminate healthy eating and physical activity programs in schools. Similar importance was mention in recent publications stating that school-based intervention approaches leads to behavioral change in terms of increase in nutritional diet consumption and less of unhealthy foods among the young generation [47, 48].

Physical education and nutritional health is not available in most high schools in Kiribati and if available are omitted during the final two years of school. Since there is always a link between health and education, schools need to reconsider the health of their students in collaboration with academic support. According to Frumkin (2006), the primary mission of school systems is to enable students to reach full potential through academic training, social values, and shared responsibilities [49]. School curriculums may need to be revised in order to enable students to reach full capacity with good health [50].

Policies concerning school food also require implementation. Students voiced that foods sold in school were affordable but are often energy-dense foods. Nearby shops are not included in school food policies and therefore selling junk foods that is readily accessible to students. The school environment that is inclusive of food preparations have huge influence on student`s diet because most of the food consumption happens in school. As Mark et al. (2014) highlighted those healthy foods in schools impact student’s lifestyle towards healthy food [51].

In addition, policies concerning physical activity in schools need consideration. Many responses request for physical education as part of high school academic learning. A review of researches concludes that addition of physical education to school curriculum will benefit students academically and physically [52] but pulling physical education away from the curriculum will not [53]. However, to implement and put into practice interventional programs would require strategies aimed at individual, community, and national levels.

### Stigma

Overweight and obesity is a complex medical condition with superfluous consequences. However, to overcome these consequences, the public finds it amusing and hilarious, resulting in decrease self-esteem and psychological stress to victimized individuals. Many students reported several attempts to exercise but are held back by shame and public comments. This shows that misconception of overweight and obesity exists among society, in fact it exists in homes, schools, communities, and even healthcare settings [54].

Additionally, psychological issues and bullying [55] arise from weight stigma as mentioned by Stevens et al. (2017) [56]. Many students expressed mostly stress and not so much depression or bullying as multiple articles has reported. This could be due to the fact that words of expression used and feelings of negativity does not tremble a student into thinking it is serious harassment but more into rationalizing that comments stated by society are all facts and requires immediate action.

### Being overweight or obese comes with high risk

Big body weight is common throughout the world and posing high risk to NCDs and its consequences of premature death and disabilities [57]. A need to combat overweight and obesity is global but searching for the most suitable approach is complex [58]. Students interviewed highlighted overweight and obesity as the chance for NCDs to develop. Peters et al. (2019) [59] and Piernas et al. (2016) [60] supported this view. This indicates that approaches to strengthen practices although immense, is recommended. The simplest and cost-effective approach as voiced by most students interviewed, is self-change with positive attitude [61].

### Limitations

This study has offered to identify factors contributing to overweight and obesity among high school students in Kiribati. However, the study sample is confined to the senior high school students that make up an average of 20% of the adolescent population [62] without considering adolescents who are not enrolled in schools. Furthermore, the sample of schools was confined to South Tarawa, the most urbanized island of Kiribati and the views of senior high schools in a more remote setting may resemble such views. It can be argued that the views of senior high school students could be comparable to those adolescents not enrolled in senior high schools and those enrolled in rural settings however, a broader study is required to validate this perspective.

The sample used in the research categories did not have even numbers. For instance, the number of participants from the participating high schools, the school form levels, the age, and the females outnumbered the males. This may create biasness in the research and have an impact on the overall perception of the Country.

## Conclusion

The main message of this research is to understand the approach to closing the gap between knowledge and practices of adolescents through use of positive role models. Taking into consideration, the cultural beliefs and social practices that devalue the influence of positive role models, it is up to an individual to choose whether to change for the better or endure the detrimental consequences of overweight and obesity. The recommendation is to recognize and understand the significance of the school-based approach in preventing overweight and obesity among adolescents where student`s time, availability of expertise, and influence of peers all exist will bring in the concept of multifactorial approach to a multifaceted cause.

## Supporting information

S1 File Questionnaire English and original version. (DOCX)

## References

[1] Wang Youfa & Lobstein Tim. Worldwide trends in childhood overweight and obesity, International Journal of Pediatric Obesity, 2006; 1:1, 11–25, doi: 10.1080/17477160600586747 17902211

[2] World Health Organization. Obesity and overweight. 2020; Retrieved from https://www.who.int/news-room/fact-sheets/detail/obesity-and-overweight

[3] Ng M., Fleming T., Robinson M., Thomson B., Graetz N., Margono C., et al. Global, regional, and national prevalence of overweight and obesity inchildren and adults during 1980–2013: a systematic analysis for the Global Burden of Disease Study 2013. Lancet (London, England), 384(9945), 766–781.2014; 10.1016/S0140-6736 (14)60460-8 PMC4624264 24880830

[4] Çelmeli G, Çürek Y, Arslan Gülten Z, Yardımsever M, Koyun M, Akçurin S, et al. Remarkable Increase in the Prevalence of Overweight and Obesity Among School Age Children in Antalya, Turkey, Between 2003 and 2015. J Clin Res Pediatr Endocrinol. 2019 Feb 20;11(1):76–81. doi: 10.4274/jcrpe.galenos.2018.2018.0108 Epub 2018 Sep 25. ; PMCID: PMC6398185. 30251957 PMC6398185

[5] Raj M., & Kumar R. K. Obesity in children & adolescents. The Indian journal of medical research, 2010; 132(5), 598–607. 21150012 PMC3028965

[6] Lee Alexandra, et al. “Social and Environmental Factors Influencing Obesity.” *Endotext* *[*Internet]., U.S. National Library of Medicine, 12 Oct. 2019, www.ncbi.nlm.nih.gov/books/NBK278977/.

[7] Russell-Mayhew S, McVey G, Bardick A, Ireland A. Mental health, wellness, and childhood overweight/obesity. J Obes. 2012;2012:281801. doi: 10.1155/2012/281801 Epub 2012 Jun 24. ; PMCID: PMC3388583. 22778915 PMC3388583

[8] Djalalinia S., Qorbani M., Peykari N., & Kelishadi R. Health impacts of Obesity. Pakistan journal of medical sciences, 2015; 31(1), 239–242. doi: 10.12669/pjms.311.7033 25878654 PMC4386197

[9] Xu Q, Zhou M, Jin D, Zeng X, Qi J, Yin L, et al. Projection of premature mortality from noncommunicable diseases for 2025: a model based study from Hunan Province, China, 1990–2016. PeerJ. 2020 Nov 3;8:e10298. doi: 10.7717/peerj.10298 ; PMCID: PMC7646306. 33194444 PMC7646306

[10] Khang YH. Burden of noncommunicable diseases and national strategies to control them in Korea. J Prev Med Public Health. Jul;46(4):155–64. doi: 10.3961/jpmph.2013.46.4.155 Epub2013 Jul 31. Erratum in: J Prev Med Public Health. 2013 Sep 2013;46(5):292. ; PMCID: PMC3740220. 23946873 PMC3740220

[11] Chan R. S., & Woo J. Prevention of overweight and obesity: how effective is the current public health approach. International journal of environmental research and public health, 2010; 7(3), 765–783. doi: 10.3390/ijerph7030765 20617002 PMC2872299

[12] Panuganti KK, Nguyen M, Kshirsagar RK. Obesity. In: StatPearls [Internet]. Treasure Island (FL): StatPearls Publishing; 2021 Jan-. Available from: https://www.ncbi.nlm.nih.gov/books/NBK459357

[13] United Nations International Children`s Emergency Fund. Adolescents Statistics. 2019; Retrieved from https://data.unicef.org/topic/adolescents/overview/

[14] Adolescent development. (2017, May 09). Retrieved from https://www.who.int/maternal_child_adolescent/topics/adolescence/development/en/

[15] National Academies of Sciences, Engineering, and Medicine; Health and Medicine Division; Division of Behavioral and Social Sciences and Education; Board on Children, Youth, and Families; Committee on the Neurobiological and Socio-behavioral Science of Adolescent Development and Its Applications; Backes EP, Bonnie RJ, editors. The Promise of Adolescence: Realizing Opportunity for All Youth. Washington (DC): National Academies Press (US); 2019 May 16.2,AdolescentDevelopment. Available from: https://www.ncbi.nlm.nih.gov/books/NBK545476/31449373

[16] Arroyo-Johnson C., & Mincey K. D. Obesity Epidemiology Worldwide. Gastroenterology clinics of North America, 45(4), 571–579. 2019; doi: 10.1016/j.gtc.2016.07.012 27837773 PMC5599163

[17] Lindberg L., Danielsson P., Persson M., Marcus C., & Hagman E. Association of childhood obesity with risk of early all-cause and cause-specific mortality: A Swedish prospectivecohortstudy. PLoSmedicine,(2019,(3),e1003078. 10.1371/journal.pmed.1003078 PMC7080224 32187177

[18] Pengpid S., Peltzer K. Childhood overweight and social correlates among school-going adolescents in Dominica and Jamaica. Afr. J. Phys. Health. Educ. Recr. Dance 2014, 20, 636–645.

[19] Chand S. S., Singh B., & Kumar S. The economic burden of non-communicable disease mortality in the South Pacific: Evidence from Fiji. Plos One, (2020).15(7). doi: 10.1371/journal.pone.0236068 32702003 PMC7377477

[20] Hagaman AK, Wutich A. How many interviews are enough to identify metathemes in multisited and cross-cultural research? Another perspective on guest, bunce, and Johnson’s (2006) landmark study. Field Methods. 2017;29(1):23–41.

[21] Sandelowski M. Sample size in qualitative research. Res Nurs Health. 1995;18(2):179–83. doi: 10.1002/nur.4770180211 7899572

[22] Ritchie J, Lewis J, Elam G. Designing and selecting samples. In: Ritchie J, Lewis J, editors. Qualitative research practice: a guide for social science students and researchers. London: Sage; 2003. p. 77–108.

[23] Bree R. T., & Gallagher G. T. Using Microsoft Excel to code and thematically analyse qualitative data: A simple, cost-effective approach. 2016; Retrieved from https://ojs.aishe.org/index.php/aishe-j/article/view/281

[24] Young M., Varpio L., Uijtdehaage S., & Paradis E. The Spectrum of Inductive and Deductive Research Approaches Using Quantitative and Qualitative Data. Academic medicine: journal of the Association of American Medical Colleges, 2020; 95(7), 1122. doi: 10.1097/ACM.0000000000003101 31833855

[25] Guba E. G., Lincoln Y Fourth generation evaluation. Newbury Park, CA: Sage. 1989.

[26] Institute of Medicine (US) Committee on an Evidence Framework for Obesity Prevention Decision Making; Kumanyika SK, Parker L, Sim LJ, editors. Bridging the Evidence Gap in Obesity Prevention: A Framework to Inform Decision Making. Washington (DC): National Academies Press (US); 2010.1, Introduction. Available from: https://www.ncbi.nlm.nih.gov/books/NBK220182/25032376

[27] Bautista-Castaño I., Sangil-Monroy M., Serra-Majem L., & Comité de Nutrición y Obesidad Infantil de la Sociedad Española de Nutrición Comunitaria Conocimientos y lagunas sobre la implicación de la nutrición y la actividad física en el desarrollo de la obesidad infantil y juvenil [Knowledge and gaps on the role of nutrition and physical activity on the onset of childhood obesity]. Medicina clinica,2004; 123(20),782–793. doi: 10.1016/s0025-7753(04)74668-0 15607072

[28] Poverty Levine J. A. and obesity in the U.S. Diabetes, 2011; 60(11), 2667–2668. doi: 10.2337/db11-1118 22025771 PMC3198075

[29] Phipps S. A., Burton P. S., Osberg L. S., & Lethbridge L. N. Poverty and the extent of child obesity in Canada, Norway, and the United States. Obesity reviews: an official journal of the International Association for the Study of Obesity, 2006; 7(1), 5–12. 10.1111/j.1467-789X.2006.00217.x 16436098

[30] Blakney A. K., Zhu Y., McKay P. F., Bouton C. R., Yeow J., Tang J., et al. Big Is Beautiful: EnhancedsaRNA Delivery and Immunogenicity by a Higher Molecular Weight, Bioreducible, Cationic Polymer. ACS nano, 2020; 14(5), 5711–5727. doi: 10.1021/acsnano.0c00326 32267667 PMC7304921

[31] Day E., Jones L., Langner R., & Bluebond-Langner M. Current understanding of decision-making in adolescents with cancer: A narrative systematic review. Palliative medicine, 2016; 30(10), 920–934. doi: 10.1177/0269216316648072 27160700 PMC5117127

[32] Diaz V. A., Mainous A. G. 3rd, & Pope C. Cultural conflicts in the weight loss experience of overweight Latinos. International journal of obesity 2005; 31(2), 328–333. 10.1038/sj.ijo.0803387 16718284

[33] Péneau S., Mekhmoukh A., Chapelot D., Dalix A. M., Airinei G., Hercberg S., et al. Influence of environmental factors on food intake and choice of beverage during meals in teenagers: a laboratory study. The British journal of nutrition, 2009; 102(12), 1854–1859. doi: 10.1017/S0007114509991280 19682398

[34] Cruess S. R., Cruess R. L., & Steinert Y. Role modelling—making the most of a powerful teaching strategy. BMJ (Clinical research ed.), 2008; 336(7646), 718–721. doi: 10.1136/bmj.39503.757847.BE 18369229 PMC2276302

[35] Mitchell J. A., Rodriguez D., Schmitz K. H., & Audrain-McGovern J. Greater screen time is associated with adolescent obesity: a longitudinal study of the BMI distribution from Ages 14 to 18. Obesity (Silver Spring, Md.), 2013; 21(3), 572–575. doi: 10.1002/oby.20157 23592665 PMC3630469

[36] Council on Communications and Media & Strasburger V. C. Children, adolescents, obesity, and the media. Pediatrics, 2011; 128(1), 201–208. doi: 10.1542/peds.2011-1066 21708800

[37] Robinson T. N. Television viewing and childhood obesity. Pediatric clinics of North America, 2001; 48(4), 1017–1025. doi: 10.1016/s0031-3955(05)70354-0 11494635

[38] Pearson N., & Biddle S. J. Sedentary behavior and dietary intake in children, adolescents, and adults. A systematic review. American journal of preventive medicine, 2011; 41(2),178–188. doi: 10.1016/j.amepre.2011.05.002 21767726

[39] Im M. H., Hughes J. N., & West S. G. Effect of Trajectories of Friends’ and Parents’ School Involvement on Adolescents’ Engagement and Achievement. Journal of research on adolescence: the official journal of the Society for Research on Adolescence, 2016; 26(4), 963–978. doi: 10.1111/jora.12247 28239244 PMC5321170

[40] Hooper L., Anderson AS., Birch J., Forster AS., Rosenberg G., Bauld L., et al. Public awareness and healthcare professional advice for obesity as a risk factor for cancer in the UK: a cross-sectional survey, Journal of Public Health, Volume 40, Issue 4, 2018; Pages797805, doi: 10.1093/pubmed/fdx145 29155951 PMC6306085

[41] National Collaborating Centre for Mental Health (UK). Challenging Behaviour and Learning Disabilities: Prevention and Interventions for People with Learning Disabilities Whose Behaviour Challenges. London: National Institute for Health and Care Excellence (UK); 2015; (NICE Guideline, No. 11.) 10, Environmental interventions. Available from: https://www.ncbi.nlm.nih.gov/books/NBK355381/26180881

[42] Guinat C., Vergne T., Jurado-Diaz C., Sánchez-Vizcaíno J. M., Dixon L., & Pfeiffer D. U. Effectiveness and practicality of control strategies for African swine fever: what do we really know? The Veterinary record, 2017; 180(4), 97. doi: 10.1136/vr.103992 27852963 PMC5293861

[43] Tremmel M., Gerdtham U. G., Nilsson P. M., & Saha S. Economic Burden of Obesity:A Systematic Literature Review. International journal of environmental research and public health, 2017; 14(4), 435. doi: 10.3390/ijerph14040435 28422077 PMC5409636

[44] Bleich S. N., Vercammen K. A., Zatz L. Y., Frelier J. M., Ebbeling C. B., & Peeters A. Interventions to prevent global childhood overweight and obesity: a systematic review. The lancet. Diabetes & endocrinology, 2018; 6(4), 332–346. doi: 10.1016/S2213-8587(17)30358-3 29066096

[45] Centeio E. E., McCaughtry N., Moore E., Kulik N., Garn A., Martin J., et al. Building healthy communities: A comprehensive school health program to prevent obesity in elementary schools. Preventive medicine, 2018; 111,210–215. doi: 10.1016/j.ypmed.2018.03.005 29548789

[46] Manios Y., Grammatikaki E., Androutsos O., Chinapaw M. J., Gibson E. L., Buijs G., et al., & ToyBox-study group A systematic approach for the development of a kindergarten-based intervention for the prevention of obesity in preschool age children: the ToyBox-study. Obesity reviews: an official journal of the International Association for the Study of Obesity, 2012; 13 Suppl 1, 3–12. 10.1111/j.1467-789X.2011.00974.x 22309061

[47] Hung L. S., Tidwell D. K., Hall M. E., Lee M. L., Briley C. A., & Hunt B. P. A meta-analysis of school-based obesity prevention programs demonstrates limited efficacy of decreasing childhood obesity. Nutrition research (New York, N.Y.), 2015; 35(3), 229–240. doi: 10.1016/j.nutres.2015.01.002 25656407

[48] Sobol-Goldberg S., Rabinowitz J., & Gross R. School-based obesity prevention programs: a meta-analysis of randomized controlled trials. Obesity (Silver Spring, Md.), 2013; 21(12), 2422–2428. doi: 10.1002/oby.20515 23794226

[49] Frumkin H. Introduction. Safe and Healthy School Environments, 2006; 3–10. doi: 10.1093/acprof:oso/9780195179477.003.0001 17448364

[50] Committee on Physical Activity and Physical Education in the School Environment; Food and Nutrition Board; Institute of Medicine; Kohl HW III, Cook HD, editors. Educating the Student Body: Taking Physical Activity and Physical Education to School. Washington (DC): National Academies Press (US); 2013; 5 Approaches to PhysicalEducationinSchools. Available from: https://www.ncbi.nlm.nih.gov/books/NBK201493/24851299

[51] Niebylski M. L., Lu T., Campbell N. R., Arcand J., Schermel A., Hua D., et al. Healthy food procurement policies and their impact. International journal of environmental research and public health, 2014; 11(3), 2608–2627. doi: 10.3390/ijerph110302608 24595213 PMC3986994

[52] Trudeau F., & Shephard R. J. Physical education, school physical activity, school sports and academic performance. International Journal of Behavioral Nutrition and Physical Activity, 2008; 5(1), 10. doi: 10.1186/1479-5868-5-10 18298849 PMC2329661

[53] Marsh H. W. Extracurricular activities: Beneficial extension of the traditional curriculum or subversion of academic goals? Journal of Educational Psychology, 1992; 84(4),553–562. doi: 10.1037/0022-0663.84.4.553

[54] Puhl R. M., & Heuer C. A. Obesity Stigma: Important Considerations for Public Health. American Journal of Public Health, 2010; 100(6), 1019–1028. doi: 10.2105/AJPH.2009.159491 20075322 PMC2866597

[55] Geel M. V., Vedder P., & Tanilon J. Are overweight and obese youths more often bullied by their peers? A meta-analysis on the relation between weight status and bullying. International Journal of Obesity, 2014; 38(10), 1263–1267. doi: 10.1038/ijo.2014.117 25002148

[56] Stevens S. D., Herbozo S., Morrell H. E., Schaefer L. M., & Thompson J. K. Adult and childhood weight influence body image and depression through weight stigmatization. Journal of health psychology, 2017; 22(8),1084–1093. doi: 10.1177/1359105315624749 26826166

[57] Islam S. M., Purnat T. D., Phuong N. T., Mwingira U., Schacht K., & Fröschl G. Nocommunicable diseases (NCDs) in developing countries: a symposium report. Globalization and health, 2014; 10, 81. doi: 10.1186/s12992-014-0081-9 25498459 PMC4267750

[58] Institute of Medicine (US) Committee on an Evidence Framework for Obesity Prevention Decision Making; Kumanyika SK, Parker L, Sim LJ, editors. Bridging the Evidence Gap in Obesity Prevention: A Framework to Inform Decision Making. Washington (DC): National Academies Press (US); Obesity Prevention Strategies in Concept and Practice. 2010; Available from: https://www.ncbi.nlm.nih.gov/books/NBK220174/25032376

[59] Peters R., Ee N., Peters J., Beckett N., Booth A., Rockwood K., et al. Common risk factors for major noncommunicable disease, a systematic overview of reviews and commentary: the implied potential for targeted risk reduction. Therapeutic advances in chronic disease, 2019;10,2040622319880392. doi: 10.1177/2040622319880392 31662837 PMC6794648

[60] Piernas C., Wang D., Du S., Zhang B., Wang Z., Su C., et al. Obesity, non-communicable disease (NCD) risk factors and dietary factors among Chinese school-aged children. Asia Pacific journal of clinical nutrition, 2016; 25(4), 826–840. doi: 10.6133/apjcn.092015.37 27702726 PMC5094276

[61] Teixeira P. J., & Marques M. M. Health Behavior Change for Obesity Management. Obesity facts, 2017 10(6), 666–673. doi: 10.1159/000484933 29237167 PMC5836178

[62] Tebakabo T. A critical examination of the impact of selected school environmental and resource factors on the academic performance of secondary school students in Kiribati [Internet]. [Suva, Fiji]: University of the South Pacific; 2007 [cited 2020 Sep 11]. Available from: http://digilib.library.usp.ac.fj/gsdl/collect/usplibr1/index/assoc/HASH1f0b.dir/doc.pdf

