# Supplementary material for: Perception of students on factors contributing to overweight and obesity among high school students in Kiribati: A qualitative study
Source: PLoS One. 2022 Jan 20;17(1):e0260900. doi: 10.1371/journal.pone.0260900 (PMC8775294; doi:10.1371/journal.pone.0260900)
Supplement: S1 File — (DOCX) [file pone.0260900.s001.docx]

**Demographics**

Name………………………………………...Date……………………………

School ……………………………

Grade level…………………………………...…

Age………………….

Gender……………………………………

Weight…………kg…………………………

Height……………meters………….……………

Residential

village………………………….

Religion………………………………………….

Parents Job………………………………….

Kava Y/N Smoke Y/N Alcohol Y/N

Play sports Y/N Regular meals Y/N Chronic illness Y/N

**General Questions**

1. Describe your understanding of overweight and obesity?
2. List the possible causes of overweight and obesity.
3. In what ways does unhealthy diet contribute to overweight and obesity?
4. In what ways does physical inactivity contribute to overweight and obesity?
5. What is the best method you think is applicable in preventing overweight an nfcd obesity?
6. Comments:

Figure 1a: Semi-structured questionnaire for students (English version)

**Rongorongom**

Aram……………………………………..

Bongin namwakaina…………………………………

Aran te reirei……………………………..

Koraki iraua…………………………..……………..

Am ririki…………………………………

Te Aine/ Te Mane……………………….

Tinebum…………………….………kg Abwakim………………………….miita

Am kaawa……………………………….

Am Aro……………………………………..………..

Aia mwakuri am karo……………………

Nangkona Y/N Moko Y/N Manging Y/N

Am takakaro…………………

Amarake nte ingabong/tawanou/tairiki Aorakim Y/N

**Titiraki**

1. Kabwarabwara am atatai nte marikarika?
2. Atong waaki aika a karika te marikarika.
3. E kanga n reke te marikarika man te amarake?
4. E kanga n reke te marikarika man te aki kakammwakuri?
5. Tera te kabanea n tamaroa ibukin totokoan te marikarika?
6. Iai am titiraki ngke kan kamataata?

Figure 1b: Semi-structured questionnaire for students (Kiribati version)
